# Metabolism and Regulation of Ascorbic Acid in Fruits

**DOI:** 10.3390/plants11121602

**Published:** 2022-06-18

**Authors:** Xianzhe Zheng, Min Gong, Qiongdan Zhang, Huaqiang Tan, Liping Li, Youwan Tang, Zhengguo Li, Mingchao Peng, Wei Deng

**Affiliations:** 1 Key Laboratory of Plant Hormones and Development Regulation of Chongqing, School of Life Sciences, Chongqing University, Chongqing 400044, China; 20117318@cqu.edu.cn (X.Z.); 202026021035@cqu.edu.cn (M.G.); 202026021016@cqu.edu.cn (Q.Z.); zhengguoli@cqu.edu.cn (Z.L.); 2 Institute of Horticulture, Chengdu Academy of Agriculture and Forestry Sciences, Chengdu 611130, China; huaqiang@gmail.com (H.T.); anzerna@126.com (L.L.); tangyouwan@126.com (Y.T.)

**Keywords:** ascorbic acid, fruit, metabolic, regulatory genes, hormones, environmental factors

## Abstract

Ascorbic acid, also known as vitamin C, is a vital antioxidant widely found in plants. Plant fruits are rich in ascorbic acid and are the primary source of human intake of ascorbic acid. Ascorbic acid affects fruit ripening and stress resistance and plays an essential regulatory role in fruit development and postharvest storage. The ascorbic acid metabolic pathway in plants has been extensively studied. Ascorbic acid accumulation in fruits can be effectively regulated by genetic engineering technology. The accumulation of ascorbic acid in fruits is regulated by transcription factors, protein interactions, phytohormones, and environmental factors, but the research on the regulatory mechanism is still relatively weak. This paper systematically reviews the regulation mechanism of ascorbic acid metabolism in fruits in recent decades. It provides a rich theoretical basis for an in-depth study of the critical role of ascorbic acid in fruits and the cultivation of fruits rich in ascorbic acid.

## 1. Introduction

Ascorbic acid (AsA), also known as vitamin C, is an important antioxidant widely found in plants. AsA is closely related to human health. A lack of AsA can lead to scurvy [1]. In addition, AsA also plays an important role in the defense of cardiovascular diseases, cataracts, cancer, aging, and other diseases [2,3]. Since the human body lacks the gene encoding L-guluronic acid-1,4-lactone oxidase, the last step in the AsA synthesis pathway, it cannot synthesize AsA [4]. Therefore, fruit is the main source of human AsA intake.

As an important reactive oxygen species scavenger, AsA plays an important role in plant resistance to biotic stress. The increase in endogenous ascorbic acid content can improve the resistance of potato to Phytophthora infestans [5]. Seed priming with salicylic acid treatment enhanced tomato resistance to Fusarium wilt [6]. In addition to biotic stress conditions, more studies have shown that AsA played an important role in abiotic stress resistance [7,8]. The reactive oxygen species produced by drought, salinity, freezing, and other abiotic stresses can be eliminated by AsA, thereby protecting cells from damage [9,10,11]. Exogenously applied AsA can alleviate the adverse effects of various abiotic stresses and promote plant growth [12,13].

AsA plays an important role in the postharvest low-temperature storage of fruits. Exogenous AsA treatment can significantly improve the cold resistance of banana fruit and strawberry fruit under low-temperature storage [14,15]. Changes in AsA content can also affect fruit ripening [16]. In addition, AsA has a certain relationship with fruit development. After reducing the AsA content in tomato fruit, the size of tomato fruit is significantly reduced [17]. Understanding the metabolism and regulatory mechanism of AsA in fruit has important guiding significance for fruit quality improvement and postharvest preservation. This paper systematically reviews the AsA metabolism and regulatory mechanism in fruits in recent decades.

## 2. Metabolic Pathway of Ascorbic Acid

**Figure 1:**
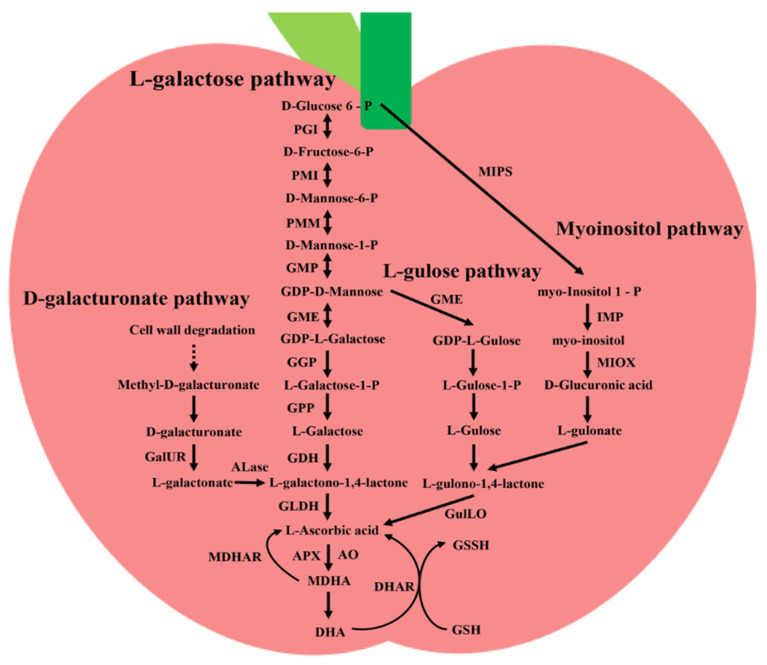
The AsA metabolic pathway in plants. The main pathways for ascorbic acid synthesis in plants are L-galactose pathway, Myoinositol pathway, L-gulose pathway, and D-galacturonate pathway. Alase, aldono-lactonase; AO, ascorbate oxidas; APX, ascorbate peroxidase; DHA, dehydroascorbic acid; DHAR, dehydroascorbate reductase; GalUR, D-galacturonate reductase; GDH, L-galactose dehydrogenase; GGP, GDP-L-galactose-phosphorylase; GLDH, L-galactono-1,4-lactone dehydrogenase; GME, GDP-D-mannose3′, 5′-epimerase; GMP, GDP-mannose pyrophosphorylase; GPP, L-galactose-1-phosphate phosphatase; GSH, glutathione; GSSH, oxidized glutathione; GulLO, L-gulono-1,4-lactone oxidase; IMP, myoinositol monophosphatase; MDHA, monodehydroascorbic acid; MDHAR, monodehydroascorbate reductase; MIOX, myoinositol oxygenase; MIPS, myoinositol phosphate synthase; PGI, phosphoglucose isomerase; PMI, phosphomannose isomerase; PMM, phosphomannomutase.

The main dietary sources of AsA for humans are fruits. The AsA contents of some fruits are organized, such as tomato, kiwifruit, orange, strawberry, carrot, sweet pepper, and so on (Table 1) [18,19,20,21,22,23,24,25,26,27,28,29,30]. Because of the important role of AsA in plants, in-depth studies have been carried out on the metabolic pathways of AsA in plants (Figure 1).

### 2.1. Biosynthesis of Ascorbic Acid

The AsA synthesis has been demonstrated to occur in the mitochondria via several proposed routes. The L-galactose pathway, also called the Smirnoff–Wheeler pathway, is the major biosynthesis pathway [31]. *Arabidopsis* is a model plant for gene function research, and most genes and enzymes involved in AsA biosynthesis have been well characterized and described. In the L-galactose pathway, D-glucose-6-phosphate is first converted to D-fructose-6-phosphate by phosphoglucose isomerase (PGI) [4]. Then phosphomannose isomerase (PMI) catalyzes the conversion of D-fructose-6-phosphate to D-Mannose-6-phosphate, which is then converted to D-mannose-1-phosphate by phosphomannomutase (PMM) [32,33]. GDP-mannose pyrophosphorylase (GMP), named *VTC1* in *Arabidopsis*, catalyzes a rate-limiting step in AsA synthesis that converts D-mannose-1-phosphate to GDP-D-mannose [34]. GMP gene family has been identified in several horticultural crops, including tomato, apple, and pear [35,36,37]. In the next step, GDP-D-mannose3′, 5′-epimerase (GME) catalyzes GDP-D-mannose conversion to GDP-L-galactose [38]. The first dedicated step in the L-galactose pathway is accomplished by GDP-L-galactose-phosphorylase (GGP), which converts GDP-L-galactose to L-galactose-1-phosphate [39]. In *Arabidopsis*, GGP is encoded by the *VTC2* and *VTC5* gene and low content of AsA is presented in *vtc2* and *vtc5* mutants [40]. Then the removal of the phosphate group by L-galactose-1-phosphate phosphatase (GPP) to L-galactose-1-phosphate produces L-galactose [41]. Although GPP in *Arabidopsis* (encoded by the *VTC4*) has phosphatase activity, AsA is still accumulated in vtc4 mutants, suggesting that GPP is not a key rate-limiting enzyme in AsA biosynthesis [42,43]. Lastly, L-galactose dehydrogenase (GDH) converts L-galactose to L-galactono-1,4-lactone, which is finally restored to AsA by L-galactono-1,4-lactone dehydrogenase (GLDH) [41,44]. Almost all enzymes involved in AsA biosynthesis are located in the cytosol, but GLDH is located in the mitochondrial inner membrane, indicating that AsA is finally synthesized in the mitochondria [45,46].

In plants, the L-galactose pathway is the major but not the only pathway leading to AsA synthesis. Studies have found that, in addition to catalyzing GDP-D-mannose to produce GDP-L-galactose, GME can also catalyze GDP-D-mannose to GDP-L-gulose [47]. In the L-gulose pathway, GDP-L-gulose is successively converted to L-Gulose-1-phosphate, followed by L-Gulose, which can be converted into L-gulono-1,4-lactone [48]. The L-gulono-1,4-lactone is finally converted to AsA by L-gulono-1,4-lactone oxidase (GulLO), a paralogous gene of GLDH [49]. Two GulLO genes in *Arabidopsis* (*AtGulLO3* and *AtGulLO5*) have been characterized [50]. However, there are almost no reports on the characterization of GulLO in fruits.

L-gulono-1,4-lactone oxidase is not only the last precursor of AsA biosynthesis in L-gulose pathway, but also the last precursor of AsA biosynthesis in the myoinositol pathway [51]. Like in animals, myoinositol can be converted to D-glucuronate by myoinositol oxygenase (MIOX) in plants [52]. It has been reported that overexpression of *AtMIOX4* can increase the AsA content in *Arabidopsis* [51]. D-glucuronate is then sequentially converted to L-gulonate and L-gulono-1,4-lactone [4].

In addition, the D-galacturonate pathway is also a potential pathway of AsA biosynthesis [48]. Methyl-D-galacturonate is one of the cell wall degradation products and can be converted to D-galacturonate by methylesterase. Then, D-galacturonate reductase (GalUR) catalyzes the conversion of D-galacturonate to l-galactonate, which is then converted to L-galactono-1,4-lactone by aldono-lactonase (Alase) [53]. However, direct evidence for the characterization of Alase in plants is still missing. Although previous studies have shown that feeding L-galactose and D-galacturonate can enhance the content of AsA in red ripened fruits of tomatoes, there is a lack of sufficient genetic evidence to illustrate this pathway in AsA biosynthesis [54].

### 2.2. Regeneration and Degradation of Ascorbic Acid

Aside from the above biosynthesis pathways, regeneration of AsA through the Foyer-Halliwell–Asada cycle is also an approach for AsA production [55]. Ascorbate peroxidase (APX) catalyzes the conversion of AsA to monodehydroascorbic acid (MDHA), which is then converted to dehydroascorbic acid (DHA) by a nonenzymatic disproportionation [3]. Ascorbate oxidas (AO) is an ascorbate oxidase, and part of AsA can also form MDHA under the catalysis of AO. Meanwhile, both MDHA and DHA can be converted to AsA by monodehydroascorbate reductase (MDHAR) and dehydroascorbate reductase (DHAR), respectively [3]. Much evidence has verified that DHAR positively controls AsA production in some horticultural crops, such as tomatoes and potatoes [56,57]. Although *MDHAR* also governs AsA regeneration, the expression level of *MDHAR* does not always coincide with AsA content. Transgenic plans are overexpressing MDHAR to decrease AsA accumulation, while knockdown of *MDHAR* gene decreases the AsA content [56,58,59]. However, overexpression of acerola MDHAR in tobacco significantly increases AsA content [60].

AsA regeneration is often closely associated with environmental stresses. AsA will be degraded through multiple pathways in apoplast when supernumerary AsA is no longer needed in plants [48]. On the one hand, DHA is hydrolyzed to 2,3-diketo-gulonic acid, which is then hydrolyzed to multiple products, including L-threarate, oxalate, and tartaric acid [61,62,63]. On the other hand, DHA can be oxidated directly to L-threarate and oxalate [64]. Moreover, DHA can also be oxidated to 4-O-oxalyl-L-threonate, which is then oxidated to L-threarate and oxalate [64]. Tartaric acid is an essential determinant of the fruit quality of the grape. Still, it cannot be detected from degradation products, indicating that oxidation of DHA is the primary process involved in AsA degradation [65].

### 2.3. Ascorbic Acid Transport

**Figure 2:**
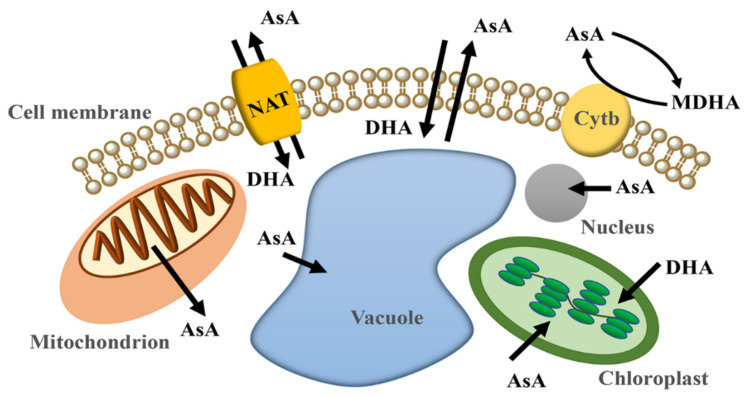
The AsA transport in plant cell. AsA is synthesized on the inner mitochondrial membrane and transported into the cytoplasm. AsA in the cytoplasm can enter organelles, such as vacuoles, chloroplasts, and nuclei, through diffusion or carriers. In addition, AsA can also be transported outside the cell membrane by simple diffusion or transport proteins. DHA in the apoplast can also enter the cell membrane and participate in the regeneration of AsA. Arrows indicate the direction of material transport. NAT, nucleobase/ascorbate transporter; Cytb, cytochrome b.

Although AsA is known to be synthesized in the mitochondrial inner membrane, AsA can also be detected in other subcellular structures, such as apoplast, chloroplast and vacuole, indicating the presence of AsA transmembrane transport [66,67,68] (Figure 2). At the outset, it is often assumed that AsA may be released from mitochondria into the cytosol via simple diffusion [69]. However, at physiological pH values, AsA has no membrane permeability [70]. Interestingly, it has been reported that mitochondrial ascorbic acid transporter from potato has unidirectional AsA transport activity, demonstrating the existence of transport proteins in mitochondria [71]. Currently, it is known that AsA is transferred from cytosol to chloroplast in a carrier-mediated manner [69]. In plants, AtPHT4;4 is the first chloroplast-localized ascorbate transporter identified from *Arabidopsis* [72]. In addition, some MDHAR and DHAR have also been shown to localize at chloroplast, suggesting that DHA can be taken up by chloroplast [73,74]. However, the mechanism of DHA uptake is still unknown. AsA utilizes concentration and pH gradients to enter the vacuole and thylakoid by passive diffusion [75]. Due to the missing AsA regenerated enzymes in the apoplast, AsA transport between protoplast and apoplast is necessary [66]. In mammals, AsA and DHA crossing the plasma membrane are mediated by Na-dependent transporters and glucose transporters [76,77]. However, the carriers that help AsA and DHA across the plasma membrane have not yet been identified [3]. Some nucleobase/ascorbate transporter (NAT) homologous genes have been identified and have functional redundancy according to the phenotype of multiple mutants. However, the regulation mechanism of *NAT* genes on AsA transmembrane transport is still unclear [78]. Furthermore, MDHA can reduce to AsA by accepting electrons from cytochrome b in the apoplast. MDHA can reduce to AsA by cytochrome b, which provides electrons from protoplast AsA oxidation [79].

AsA biosynthesis occurs in various tissues of plants, but some evidence shows that AsA has long-distance transport between tissues. AsA biosynthesis-related enzymes can be detected in the phloem of *Cucurbita pepo* fruits, indicating that AsA may be transported to other tissues through the phloem [80]. By labeling AsA with C-14, Franceschi and Tarlyn find that AsA can be transported from leaf to flower and root tip [81]. In addition, the AsA in the phloem and tubers of potato will be enriched with the increase in the AsA content in the leaves [82]. The inter-tissue transport of AsA still needs more evidence to support it.

## 3. Transcriptional Regulation of Ascorbic Acid Metabolic Genes in Fruit

Ascorbic acid is enriched in fruit and is closely related to fruit development and resistance. Changing the content of ascorbic acid in fruit is of great significance for improving fruit quality. Tomato, strawberry, kiwifruit, citrus, and other fruits are rich in ascorbic acid, with complete genome information and a mature genetic transformation system, which are the main objects of research related to the regulation of ascorbic acid content. For example, there were significant differences in the expression levels of AsA synthesis and metabolism-related genes in citrus fruits with different ascorbic acid contents [83,84]. It is of great significance to improve the ascorbic acid content in fruit by changing the expression of genes related to AsA synthesis and metabolism.

Substrate feeding experiments have shown that AsA synthesis in tomato leaves mainly depends on the L-galactose pathway, while the L-galactose pathway, the D-galacturonate pathway, and the myo-inositol pathway are all involved in AsA synthesis in tomato fruit [35]. Many genes encoding key enzymes in the AsA metabolism pathway have been identified. The content of AsA is significantly changed in fruits when the expression of these genes is altered through genetic engineering (Table 2). GMP is a key rate-limiting enzyme in the L-galactose pathway. There are four members of the GMP family in tomato (*SlGMP1*-*SlGMP4*). Upregulation or downregulation of *SlGMP3* expression in tomato fruit can significantly increase or decrease the content of AsA [85]. Overexpression of tomato *SlGME1* and *SlGME2* increase AsA content to 1.60- and 1.24- fold in ripe red fruit, respectively [86]. However, silencing *SlGME1* and *SlGME2* alone does not affect AsA content in tomato fruit [87]. Conversely, simultaneous silencing of both *SlGMEs* genes significantly reduces AsA content in tomato fruit, suggesting a functional redundancy between *SlGME1* and *SlGME2* [38]. Overexpression of *GGP* significantly increases AsA content in tomato leaves but does not affect AsA content in fruit [35]. However, studies have also shown that the AsA content in leaves, flowers, and fruits is significantly reduced in tomato *SlGGP1* mutants [88,89]. Heterologous expression of the *GGP* gene of kiwifruit in tomato and strawberry results in a twofold increase in AsA content in the fruits [90]. In addition, overexpression of *AcGGP3* can significantly increase the AsA content in leaves and fruits of kiwifruit, indicating that the role of GGP in the AsA synthesis pathway is tissue-specific in different plants [91,92]. GPP catalyzes the dephosphorylation of L-galactose-1-phosphate to L-galactose, an essential part of the L-galactose pathway. However, no significant changes in AsA content are found in the fruits of the *GGP* overexpression line and the *GGP* × *GPP* pyramiding lines [35]. A recent study by Zheng et al. indicates that overexpression of *SlIMP3*, a gene encoding a bifunctional enzyme with GPP and IMP activities, significantly increased AsA content in tomato leaves, stems, and fruits [93]. Studies on the functions of GalDH and GLDH in AsA synthesis are limited. The only research shows that GLDH has no significant effect on AsA accumulation in tomato fruits [17]. L-gulono-γ-lactone oxidase (*GLOase*) is the homologous gene of *GLDH* in animals. A 1.5-fold increase in AsA levels is found in tomato fruit heterologously expressing rat GLOase [94]. In the myo-inositol pathway, MIOX is involved in the production of D-glucuronate. Heterologous expression of *AtMIOX* increases the AsA content of tomato fruit by 1.4-fold [95]. Munir et al. identifies five MIOX (*MIOX1*~*MIOX5*) in tomatoes, and overexpression of *MIOX4* significantly increases the AsA content in leaves and red fruits [96]. *GalUR* is downstream of *MIOX*, which catalyzes the conversion of D-glucuronate to L-gulonate. Overexpression of *GalUR* from strawberries results in a 1.2- to 2.5-fold increase in AsA levels of tomato fruits [97]. Pepper fruits are rich in ascorbic acid, and the ascorbic acid content increases with fruit ripening [98]. Although the L-galactose pathway is the main AsA synthesis pathway in pepper fruits and leaves, the transcript levels of genes encoding key enzymes in the AsA biosynthesis pathway were not positively correlated with AsA concentrations in pepper pericarp [99].

In addition to changing the expression of genes related to the AsA synthesis pathway by targeting, the AsA content in fruits can also be regulated by changing the genes associated with AsA degradation and regeneration. For example, AsA content is increased 1.4- to 2.2-fold in the fruits of *APX*-downregulated tomato lines [100]. Downregulation of *AO* gene expression in tomatoes can also increase AsA content in fruit [101]. There are two SlDHARs (*SlDHAR1* and *SlDHAR2*) in tomato. Overexpression of *SlDHAR1* significantly increases the AsA content in tomato leaves and red ripe fruits. In contrast, overexpression of *SlDHAR2* only increases the AsA content in leaves, indicating that the regeneration of AsA in fruits is mainly regulated by *SlDHAR1* [102]. The upregulated [101] *SlMDHAR* expression leads to a 0.7-fold decrease in AsA content in tomato fruits but has no effect on AsA content in tomato leaves [56]. These results suggest that *SlDHAR1* and *SlMDHAR* play regulatory functions in different processes of tomato fruit ripening but are not the main limiting factor in leaves. During citrus fruit ripening, the changes in ascorbic acid content were not completely consistent with the expression of ascorbic acid metabolism-related genes, but were regulated by complex and specific interactions of synthesis and recycling-related genes [103,104].

## 4. Regulatory Genes That Control Ascorbic Acid Accumulation

Aside from genetic engineering to control the expression of genes related to AsA metabolism, plants have a variety of transcriptional and translational regulatory mechanisms. The transcription factors can bind to gene promoters and control the expression of target genes at the transcriptional level. There have been some preliminary studies on the regulation of AsA accumulation in fruits by transcription factors (Table 3). *SlICE1*, a bHLH transcription factor, is closely related to chilling resistance in tomatoes. Overexpression of *SlICE1* in tomatoes increases the AsA content in the ripe red fruit of tomato and improves the resistance of tomato fruit to low-temperature stress [105]. In tomato L1L4 transcription factor mutants, AsA content is significantly increased, suggesting that L1L4 may negatively influence AsA synthesis [106]. Heterologous expression of *Arabidopsis* brassinosteroid response transcription factor Brassinazole resistant 1 (*BZR1-1D*) in tomato can significantly increase AsA content in fruit [107]. It is found that MADS-box and CCAAT motifs are enriched in the promoters of tomato AsA synthesis-related genes, indicating that a single transcription factor may regulate multiple AsA synthesis genes [108]. Overexpression of the banana *MaMADS7* transcription factor in tomatoes significantly increases the AsA content in the fruit [109]. The CCAAT-box transcription factor SlNFYA10 in tomatoes can bind to the promoter of *SlGME1* and negatively regulate the expression of *SlGME1* and the level of AsA in leaves and fruits [110]. An HD-Zip I family transcription factor SIHZ24 is identified in tomato, which binds to the promoter of *SlGMP3*, activates the expression of *SlGMP3*, and then positively regulates the accumulation of AsA [111]. In addition, *SlHZ24* can bind to the promoters of *GME2* and *GGP* and regulate the accumulation of AsA by multiple targets [111]. *MdERF98* activates the expression of *MdGMP1*, thereby promoting the synthesis of AsA in apple [36]. Transient expression of *AcERF91* in kiwifruit can increase AsA content. At the same time, *AcERF91* can bind to the promoter of *AcGGP3* and activate the transcription of *AcGGP3* [112]. Pattern regulation of the *AceMYBS1* gene indicated that *AceMYBS1* positively regulates AsA accumulation in kiwifruit [92]. In addition, AceMYBS1 can also bind to the *AceGGP3* promoter and activate its expression, indicating that multiple transcription factors may simultaneously regulate the expression of a single AsA synthesis-related gene [92]. In addition, some transcription factors that regulate AsA degradation and regeneration are also identified. MdMYB1 can bind to the promoter of *MdDHAR*, activate the expression of *DHAR*, and then increase the content of AsA in apples [113]. MsSCL26.1 can bind to the P1 region of the *MsMDHAR* promoter and activate the transcription of *MsMDHAR*, thereby inhibiting the synthesis of AsA in the apple [114]. After transient expression of *MYB*, *NAC*, and *ZIF* transcription factors in tomato, AsA metabolism-related genes, such as *GMP2*, *GalUR*, *AO2*, and *APX6*, are significantly upregulated [115]. Transcript levels of *CaMYB16* gene and *GLDH* in pepper fruit are highly correlated, indicating that *CaMYB16* may be involved in ascorbic acid synthesis in pepper fruit [116].

The research on the regulatory mechanism of AsA accumulation at the protein level is still minimal (Table 3). AcESE3 and AcMYBR can interact with AcGGP3 to regulate AsA synthesis in kiwifruit [91]. As a result of *MdAMR1L1* overexpression or silencing in apples, a negative relationship between Asc levels and *MdAMR1L1* is detected [36]. MdAMR1L1 protein stimulated MdGMP1 degradation through ubiquitination, inhibiting AsA biosynthesis at a post-translational level [36].

In addition to the genes that can encode proteins, there are many noncoding RNAs in the plant genome. In plant development, the function of small ncRNAs, such as microRNAs and small interfering RNAs, has been extensively studied in the past decade. In total, 118 differentially expressed lncRNAs (DE-lncRNAs) and 32 differentially expressed microRNAs are identified during seabuckthorn fruit development [117]. These DE-lncRNAs are particularly enriched in the biosynthesis of AsA, carotenoids, and flavonoids. A miRNA, *mdm-miR171i*, is identified in the apple that explicitly targets and degrades MsSCL26.1, thereby enhancing AsA accumulation (Table 3) [114].

## 5. Effects of Hormones on the Ascorbic Acid Accumulation in Fruits

Classical plant hormones mainly include auxin, cytokinin, gibberellin (GA), ethylene, abscisic acid (ABA), salicylic acid (SA), jasmonic acid (JA), and brassinolide, which play an important regulatory role in fruit ripening and nutritional quality. Many studies have used phytohormones to regulate the accumulation of AsA in fruits. Application of GA3 to plum fruit delays the decrease in AsA concentration and reduces flesh browning development during storage at low temperature [118]. The combined treatments of GA3 and phenylurea significantly delay the losses in AsA contents and suppress fruit softening of harvested banana fruit [119]. GA3 (50 ppm) + 1-methyl-cyclopropane-treated strawberry fruits increase the retention of vitamin C over their shelf life compared to the control group [120]. Exogenous gibberellin and ethylene treatments can also significantly increase ascorbic acid content in citrus fruits [121]. According to a recent study, 6-benzylaminopurine and kinetin increase the AsA content of strawberry fruits by 33.96% and 27.22%, respectively, compared to the control [122].

The plant growth regulator ABA plays a role in fruit ripening. Abscisic acid and ethylene regulate AsA synthesis through antagonism [123]. It has been found that ABA alters the AsA redox state at the early stages of fruit development and more than doubles AsA levels at the end of fruit ripening in red raspberry (*Rubus idaeus* L.) [124]. Strawberry had a 1.6-fold increase in ascorbic acid content after treatment with 1 μM abscisic acid [125]. La(NO3)3 treatment can induce ABA synthesis in strawberry fruit [126]. At the same time, La(NO3)3 increases the activities of DHAR, MDHAR, and GalLDH and decreases the activities of APX and AOO, resulting in increased AsA content in strawberry fruit [126]. Tungstate is an ABA synthesis inhibitor. Tungstate treatment can activate the expression of GR, MDHAR, and GalLDH, while inhibiting the expression of DHAR and APX, thereby inhibiting the accumulation of AsA in strawberry fruit [126].

SA is a vital phytohormone involved in regulating plant resistance to various biotic and abiotic stresses. Treatment with 2 mM SA can maintain the level of AsA and reduce the chilling injury of pomegranate fruit during low-temperature storage [127]. SA treatment also significantly delays the reduction in AsA content in plum, cornelian cherry, pineapple, pear, and strawberry fruits during low-temperature storage and improves the resistance of fruits to low-temperature stress [128,129,130,131,132]. Under the combined treatment of 1.0 mM SA and 2% chitosan, the content of AsA in lychee fruit is maintained at a high level [133]. In addition, using chitosan combined with SA treatment can effectively increase the AsA content of grapefruit fruit and activate the disease resistance to green mold [134].

In addition to SA, JA is also believed to play a variety of essential roles in regulating stress responses and plant growth [135]. MeJA treatment can significantly increase the AsA content in star fruit, blueberry, and pineapple fruits and delay the quality decline of fruits under low-temperature storage [136,137,138]. Postharvest cherry tomato fruits treated with MeJA contain significantly higher AsA and carotenoids, especially lycopene [139]. MeJA treatment could inhibit the activity of AO and increase the activity of DHAR, thereby increasing the content of AsA in loquat fruit and delaying the occurrence of internal browning caused by chilling injury [140]. MYC2 acts as a regulatory center of JA signaling and is involved in cold resistance in many horticultural crops [141]. *MYC2* participates in the AsA-GSH cycle and regulates the cold tolerance of tomato fruit by regulating the accumulation of AsA [142]. Treatment with 0.25 mM MeJA can significantly increase ‘Kumato’ tomato fruit yield and AsA content [143]. The treatment of pomegranate and blueberry fruits with MeJA can also increase the AsA content and total antioxidant activity in the fruits and improve the fruits’ antioxidant capacity and storage stability [144,145]. In addition, MeJA pretreatment effectively prevents wound-induced loss of AsA and organic acids and the deterioration of the flesh color of freshly cut pitaya fruits [146].

Melatonin (MT), a hormone found in the pineal gland, has also been found in plants. As a new research-hot phytohormone, melatonin plays a vital role in scavenging reactive oxygen species and improving the resistance of plants to environmental stress [147]. Little research has been conducted on the relationship between melatonin and AsA. A 100 μM melatonin treatment increases the levels of AsA and delays senescence in sweet cherries [148]. After treatment of pomegranate fruit with 100 μM melatonin, the activities of APX and GR are increased and the activity of AAO is decreased, resulting in a higher accumulation of AA and GSH and improved resistance of the fruit to cold [149]. As well as being essential antioxidants, there may also be a deeper relationship between melatonin and AsA.

## 6. Regulation of Ascorbic Acid Accumulation by Environmental Factors

**Figure 3:**
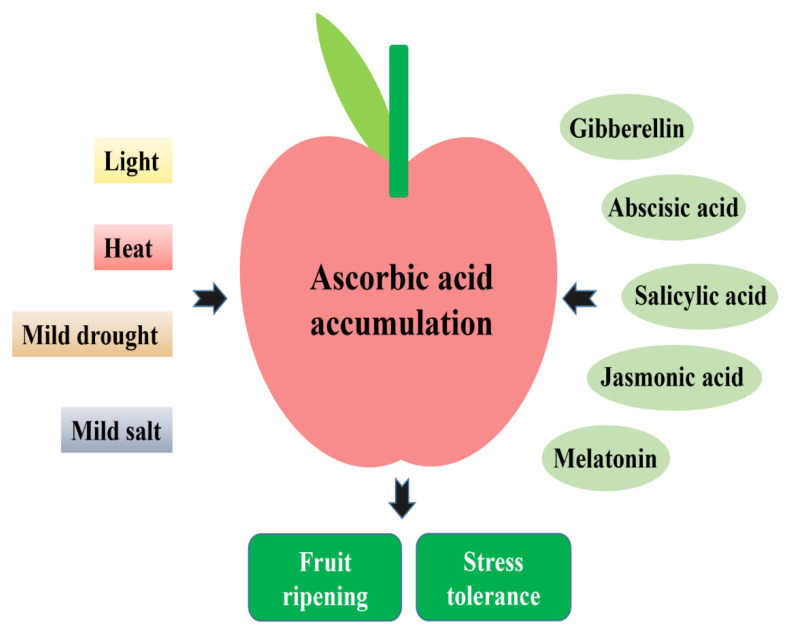
Effects of hormones and environmental factors on the accumulation of AsA in fruits. Light, heat, mild drought, and mild salt can stimulate the accumulation of ascorbic acid in the fruit. In addition, phytohormones, such as gibberellin, abscisic acid, salicylic acid, jasmonic acid, and melatonin, can also regulate the accumulation of ascorbic acid in fruits. These environmental factors and phytohormones affect fruit ripening and stress resistance by regulating the accumulation of ascorbic acid in fruits.

Environmental factors, such as temperature, light, and water, profoundly impact fruit development and nutritional quality. The accumulation of AsA in the fruit helps to improve the nutritional value of the fruit and improve the resistance of the fruit to various environmental stresses. Studying the effect of environmental factors on the accumulation of AsA in fruits has essential theoretical and practical significance for cultivating high-quality fruits rich in AsA (Figure 3). Light and AsA accumulation in fruit are closely related. After shading treatment of tomatoes, fruit ripening is delayed and AsA content is also significantly decreased [150,151]. Similarly, light affects the AsA content of citrus fruits [121,152]. The research on the mechanism of light-induced AsA synthesis is still very preliminary. The D-mannose/L-galactose pathway produces a majority of AsA in plants. However, although light induces tomato fruit ripening and AsA accumulation, the carbohydrate levels in the fruit did not change significantly. Changes in carbohydrate content in fruit also did not affect the light-induced AsA synthesis, indicating that light-induced AsA synthesis in tomato fruit is independent of carbohydrates in vivo [153]. In addition, light induces the expression of critical genes in the D-mannose/L-galactose pathway, while inhibiting the expression of genes related to AsA degradation, thereby enhancing the synthesis of AsA in tomato fruit [154].

Long-term low-temperature storage can damage the quality of the fruit. With the increase in low-temperature storage time, low-temperature damage occurs in mango fruit, and the content of antioxidants, such as AsA, increased [155]. However, a recent study showed that a 1-min heat treatment at 55 °C can significantly reduce the damage of red bell pepper fruits during low-temperature storage. Preheating can substantially increase the content of AsA and glutathione during refrigeration [156]. Mild drought and salt stress can also increase the content of anthocyanin and AsA in strawberry fruit without affecting the yield [157]. These studies show that moderate environmental stress stimulation has a good application prospect in improving fruit quality.

## 7. Conclusions

AsA plays an important role in regulating fruit quality and stress resistance. Although most genes related to ascorbic acid synthesis and metabolism have been found in model plants, such as *Arabidopsis*, studies on fruits are very limited. The discovery of the functions of ascorbic acid anabolism-related genes in fruits has important guiding significance for the use of genetic engineering technology to change the AsA content in fruits. At the same time, the regulatory mechanism of AsA accumulation in fruits is still lacking, and more regulators at the transcriptional and post-translational levels need to be discovered. Plant hormones, such as gibberellin, ethylene, salicylic acid, jasmonic acid, and melatonin, can all affect the accumulation of AsA in fruits, but the regulatory mechanism is still unclear and needs further study. In addition, light and moderate environmental stress are beneficial to the accumulation of ascorbic acid in fruit. Using different environmental factors to increase the content of AsA in fruit also has important application value for fruit quality improvement.

## Figures and Tables

**Table 1:** AsA content in fruits of different cultivars.

Common Name	Cultivar	Content of AsA (mg/100 g FW)	Reference
Tomato	*Solanum pennellii*	4.40–17.61	[18]
	Cherry	31.98–42.68	[19]
	Monika	41.41–53.10	[19]
	Isabella	41.19–48.65	[19]
	HLY	5.58–18	[20]
	Rio Grande	6.78–10.8	[20]
Kiwifruit	Hayward	51.3–79.7	[21]
	Jiangxi 79-1	53.8–93.6	[21]
	Awaji	22.2–28.8	[21]
	Kosui	31.5–50.3	[21]
Strawberry	Praratchatan	63.73–72.57	[22]
	Sagahonoka	56.8	[23]
	Sugyeong	108.1	[23]
Pear	Banda	10.2	[24]
	Limon	10.1	[24]
	İncir	4.4	[24]
Orange	Mandarin	41.3	[25]
	Hamlin	62.7	[25]
	Salustiana	56.8	[25]
Watermelon	Crimson sweet	11.86–15.27	[26]
	Giza	15.58–30.45	[26]
	Dumara	10.84–23.34	[26]
Lemon	Ovale di Sorrento	29.91	[27]
	Sfusato Amalfitano	27.71	[27]
	Femminello Cerza	26.90	[27]
	Femminello Adamo	26.69	[27]
Apple	*M. pumila* ‘Saiwaihong’	0.96 ± 0.06	[28]
	Yantai Fuji No. 3	0.37 ± 0.09	[28]
	Xinshiji	0.25 ± 0.05	[28]
	Liuyuehong	0.20 ± 0.04	[28]
	Gala	0.10 ± 0.06	[28]
	Starkrimson	0.05 ± 0.02	[28]
Grape	Vrboska	0.46	[29]
	Jakubské	0.41	[29]
	Perlette	0.36	[29]
Pepper	Segana	71.99	[30]
	Catas	68.56	[30]
	Domba	61.22	[30]

**Table 2:** Relationship between expression patterns of AsA metabolism-related genes and AsA accumulation in fruits.

Common Name	Gene Name	Gene Source	Strategy	Change of AsA Content	Fold Change	Reference
tomato	GMP3	tomato	overexpression	up	1.1–1.6	[85]
	GMP3	tomato	RNAi	down	1.3–2.4	[85]
	GME1	tomato	overexpression	up	1.6	[86]
	GME1	tomato	RNAi	no change	0	[87]
	GME2	tomato	overexpression	up	1.2	[86]
	GME2	tomato	RNAi	no change	0	[87]
	GME1 × GME2	tomato	RNAi	down	0.2–0.6	[38]
	GGP	tomato	overexpression	no change	0	[35]
	GGP	tomato	mutant	down	0.5	[88,89]
	GGP	kiwifruit	overexpression	up	2.0	[90]
	GGP × GPP	tomato	overexpression	no change	0	[35]
	SlIMP3	tomato	overexpression	up	0.3–0.6	[93]
	SlIMP3	tomato	antisense	down	0.3–0.7	[93]
	GLDH	tomato	RNAi	no change	0	[17]
	GLOase	rat	overexpression	up	1.5	[94]
	MIOX	*Arabidopsis*	overexpression	up	1.4	[95]
	MIOX4	tomato	overexpression	up	1.5–2.3	[96]
	GalUR	strawberry	overexpression	up	1.2–2.5	[97]
	APX	tomato	RNAi	up	1.4–2.2	[100]
	AO	tomato	RNAi	up	0.3	[101]
	DHAR1	tomato	overexpression	up	0.4	[102]
	DHAR2	tomato	overexpression	no change	0	[102]
	MDHAR	tomato	overexpression	up	0.7	[56]
kiwifruit	GGP3	kiwifruit	overexpression	up	2.0–6.4	[91,92]
strawberry	GGP	kiwifruit	overexpression	up	2.0	[90]

**Table 3:** Regulation of AsA metabolism-related genes at the transcriptional and protein levels.

Common Name	Gene Name	Gene Source	Target Gene	Combination	Effect	Reference
tomato	ICE1	tomato	not described	not described	Positively regulate the accumulation of AsA	[105]
	L1L4	tomato	not described	not described	Negatively regulate the accumulation of AsA	[106]
	BZR1-1D	*Arabidopsis*	not described	not described	Positively regulate the accumulation of AsA	[107]
	MADS7	banana	not described	not described	Positively regulate the accumulation of AsA	[109]
	NFYA10	tomato	GME1	protein-DNA	Negatively regulate the expression of GME1 and the accumulation of AsA.	[110]
	HZ24	tomato	GMP3	protein-DNA	Positively regulate the expression of GMP3 and the accumulation of AsA.	[111]
	HZ24	tomato	GME2	protein-DNA	Positively regulate the expression of GME2 and the accumulation of AsA.	[111]
	HZ24	tomato	GGP	protein-DNA	Positively regulate the expression of GGP and the accumulation of AsA.	[111]
apple	ERF98	apple	GMP1	protein-DNA	Positively regulate the expression of GMP1 and the accumulation of AsA.	[36]
	MYB1	apple	DHAR	protein-DNA	Positively regulate the expression of DHAR and the accumulation of AsA.	[113]
	SCL26.1	apple	MDHAR	protein-DNA	Positively regulate the expression of MDHAR and negatively regulate the accumulation of AsA.	[114]
	AMR1L1	apple	GMP1	protein-protein	Stimulate GMP1 degradation and negatively regulate the accumulation of AsA.	[36]
	mdm-miR171i	apple	SCL26.1	RNA-DNA	Stimulate SCL26.1 degradation and positively regulate the accumulation of AsA.	[114]
kiwifruit	ERF91	kiwifruit	GGP3	protein-DNA	Positively regulate the expression of GGP3 and the accumulation of AsA.	[112]
	MYBS1	kiwifruit	GGP3	protein-DNA	Positively regulate the expression of GGP3 and the accumulation of AsA.	[92]
	ESE3	kiwifruit	GGP3	protein-protein	Positively regulate the accumulation of AsA	[91]
	MYBR	kiwifruit	GGP3	protein-protein	Positively regulate the accumulation of AsA	[91]

## Data Availability

Not applicable.
